# Orthorexia and Eating Disorders in Adolescents and Young Adults: A Systematic Review

**DOI:** 10.3390/children9040514

**Published:** 2022-04-06

**Authors:** Panagiota Skella, Maria Eleni Chelmi, Eleni Panagouli, Anastasia Garoufi, Theodora Psaltopoulou, George Mastorakos, Theodoros N. Sergentanis, Artemis Tsitsika

**Affiliations:** 1 Strategies of Developmental and Adolescent Health, School of Medicine, National and Kapodistrian University of Athens, 115 28 Athens, Greece; panagiota.skella@gmail.com (P.S.); m_b_x_m@hotmail.com (M.E.C.); elenpana@med.uoa.gr (E.P.); angaruf@med.uoa.gr (A.G.); tpsaltop@med.uoa.gr (T.P.); tsergentanis@yahoo.gr (T.N.S.); 2 Clinical Psychopathology, University of Macedonia, 546 36 Thessaloniki, Greece; 3 Department of Clinical Therapeutics, Alexandra Hospital, School of Medicine, National and Kapodistrian University of Athens, 115 28 Athens, Greece; 4 Unit of Endocrinology, Diabetes Mellitus and Metabolism, Aretaieion Hospital, School of Medicine, National and Kapodistrian University of Athens, 115 28 Athens, Greece; gmastorak@med.uoa.gr; 5 Department of Public Health Policy, University of West Attica, 122 43 Athens, Greece

**Keywords:** orthorexia, anorexia, bulimia, eating disorders, mental health, adolescents, young adults

## Abstract

Background: In recent years a new term in the field of eating behaviors has emerged, namely “orthorexia”. This behavior is associated with significant dietary restrictions and omission of food groups. The aim of the present study is to estimate the possible correlations between orthorexia and eating disorders in young adults and adolescents. Methods: A systematic review of related articles in PubMed, Google Scholar, and PsycInfo was conducted up to 30 June 2021. Results: A total of 37 studies (16,402 subjects) were considered eligible for this systematic review. Significant correlations were observed in most of the studies between orthorexia and eating disorders. However, the majority of studies adopted a cross-sectional design. Conclusions: An association between and eating disorders emerged. Prospective studies seem necessary to investigate associations and succession of orthorexia and eating disorders over time.

## 1. Introduction

Orthorexia comes from the Greek words “orthos” which means “proper” or “correct” and “orexis” which means “appetite” [1]. Eating-related behaviors that pertain to a fixation to eat healthy, a consumption of biologically pure foods regarding the personal benefits of healthy eating, describes orthorexia [2], also known as “orthorexia nervosa”. Orthorectics tend not to consume food that has been processed with pesticides, herbicides or artificial substances, and they are highly worried about the techniques and materials involved in food preparation [2]. The prevalence of orthorexia among youth and young adults is estimated as high as 27% [3]; however, there are discrepancies between the results of various studies and prevalence in this age group can be found from 6.9% [4] to 45.5% [5], whereas studies performed in the more recent years tend to show higher prevalence of orthorexia in adolescents and young adults, than those conducted years ago.

It has been postulated that orthorectic individuals are engaged in four stages of daily behavior. Stage one includes excessive thoughts considering food consumption for a specific day and the following days. Next stage involves the excessive control of collecting food products and ingredients. The third stage pertains to very careful meal preparation which is compliant with healthy eating philosophy. The last stage entails following sentiment of accomplishment or failure, depending on the perceived outcomes of all previous stages [6].

Orthorexia is not yet recognized as a mental disorder and thus it is not included among eating disorders (EDs) [7]. Moreover, it is not categorized as an obsessive-compulsive disorder (OCD) neither to DSM-IV TR [7] or DSM-5 [8]. A debate exists in the available literature, whether orthorexia should be classified as a behavioral disorder, due to the compulsion to eat only “healthy” food which could be considered as a repeated behavior. Nevertheless, some diagnostic criteria have been proposed, dealing with the restrictions of food; according to them orthorexia should be considered when subjects are not concerned with the quantity of food or body image issues and do not want to lose weight [7].

A core similarity between orthorexia and the most common eating disorders, anorexia and bulimia nervosa, pertains to the fact that food plays an important role in the individuals’ lives and an overlapping has been observed between them [9]. Perfectionism traits, such as the consistency to the dietary routine and the feelings of culpability after failing to retain food restrictions, are both featured in individuals with anorexia and orthorexia [10]. Both EDs and orthorexia share a lack of pleasure linked to food consumption and by controlling the food intake, individuals with those conditions seem to feel able to control their life [11]. Patients with anorexia nervosa or bulimia nervosa are more worried about the quantity of food consumption [12], while patients with orthorexia are concerned about the quality and the purity of food [10]. Orthorexia may be the outcome of several risk factors acting at the same time like most other eating disorders [13].

Taking the above into account, the purpose of the present systematic review is to examine the existence of possible correlations between orthorexia and eating disorders in young adults and adolescents.

## 2. Materials and Methods

### 2.1. Literature Search Strategy

A systematic literature search was performed on 30 June 2021 in PubMed, PsycINFO and Google Scholar databases. The systematic review protocol was registered in the School of Medicine, National Kapodistrian University of Athens (registration number: 29738/14-05-2021) and is available upon request. Various search terms were used, specifically combinations of the following terms as a search algorithm: (orthorexia) AND (adolescents OR “young adulthood” “young adults” OR “young adulthood”) AND (anorexia OR bulimia OR “eating disorder”). Additionally, a thorough search of the reference lists of the considered eligible studies as well as those of relevant reviews was searched to identify further eligible reports.

### 2.2. Inclusion Criteria

Inclusion criteria encompassed the following:

1. Reports on young adults up to 30 years old and adolescents of any age. Studies which were based on adolescents/young adults were separately presented in our systematic review from those including a subgroup of admixture between young adults and older individuals.
2. Presentation of data about possible correlations between orthorexia and eating disorders (AN and BN); the latter defined according to DSM 5 and ICD 10.
3. No restrictions were posed considering diagnostic tools of eating disorders.
4. Considering study design, prospective cohorts, case-control and cross-sectional studies were included.
5. Only articles written in English were included.

No restrictions were posed regarding publication year. All article titles and abstracts were screened by authors working in pairs, blindly to each other. 

### 2.3. Exclusion Criteria

Case reports, animal studies, review articles, medical hypotheses, studies not disclosing age groups, as well as studies looking for a correlation between orthorexia and gender, BMI or dietary patterns in general, were excluded.

### 2.4. Quality Assessment of Included Studies

The quality of studies was rated with the Newcastle–Ottawa scale, which was adapted for cross-sectional studies [14].

### 2.5. Data Collection Process and Data Extraction

Authors worked in pairs, blindly to each other and extracted all the relevant data according to the inclusion criteria, as described above. In any case of disagreement, team consensus was followed. Collected data were categorized into tables and in any case the type of study, country, study period, study design, sample size, gender and age distribution, sample type (entirely adolescents or young adults or admixture), confounders, definitions of eating habits and orthorexia, associations studied in relation to orthorexia and potential confounding factors assessed were included.

### 2.6. Compliance with Ethics Guidelines

This article is based on the results of previously conducted studies. The study was performed in accordance with the Preferred Reporting Items for Systematic Reviews and Meta-Analysis (PRISMA) guidelines [15].

## 3. Results

### 3.1. Study Characteristics

**Figure 1:**
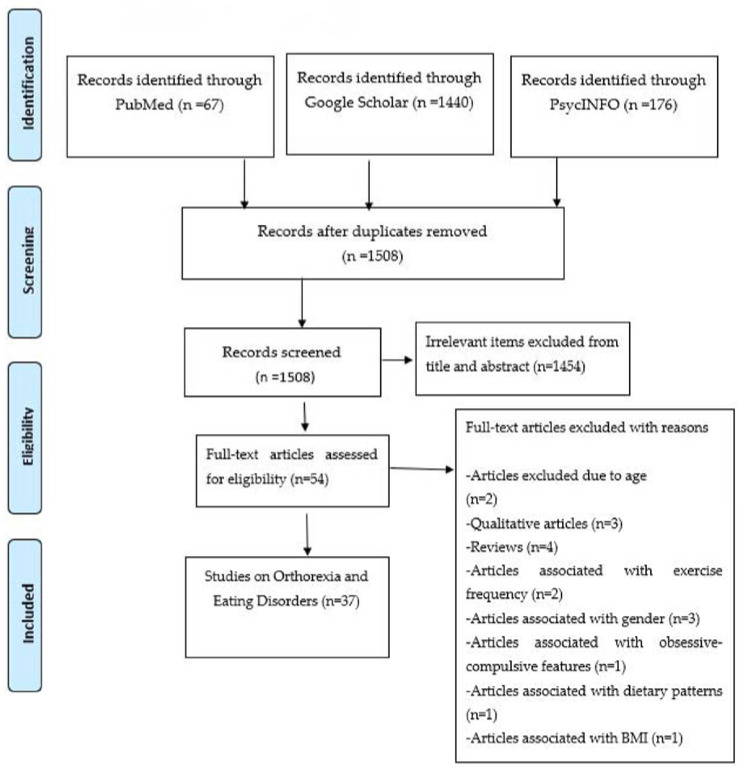
PRISMA flow diagram—selection of studies.

The literature search led to a sum of 1508 results, after removing the duplicates; of them 1454 were considered irrelevant according to title and abstract, while 54 full-texts were evaluated. Among the latter, 12 were excluded with reasons and a total of 37 studies (16,402 subjects) were finally included [2,3,11,16,17,18,19,20,21,22,23,24,25,26,27,28,29,30,31,32,33,34,35,36,37,38,39,40,41,42,43,44,45,46,47,48,49]. The PRISMA flowchart is presented in Figure 1. Table 1 shows the features and findings of included studies, as well as quality assessments; the majority of studies were rated as of low quality.

### 3.2. Assessment Tools—Observed Correlations

A number of different instruments (ORTO, EAT, EDI) were used in every study in order to define orthorexia and EDs, as presented in Table 1.

According to the majority of eligible studies, an association between orthorexia and eating disorders was reported. Specifically, Agapoyan [16], Arslantas [17], and Haddad [18] identified a significant negative correlation (*p* < 0.05) between the EAT-40 and ORTO-11 scores; higher scores in EAT-26 showed more eating pathology and lower scores in ORTO-11 and ORTO-15 showed more tendency for orthorexia. Likewise, Mahesh Shah’s study examined the individual’s scores on measures of orthorexia, eating disorder, and some other parameters, confirming the significant negative correlation between disordered eating behaviors and orthorexia nervosa (r = −0.33, *p* < 0.01) [19]. In 2017, Okumuşoğlu using a different questionnaire to measure eating disorders and the Orto-11, found a negative correlation with eating disorder tendencies which were measured by REZZY scores (r = −0.328, *p* = 0.01) [20]. McInerney-Ernst collected data from self-reported responses, indicating that orthorexia shares important characteristics with established eating disorders [21]. Self-reported disordered eating patterns significantly predicted ON symptoms [21].

Bóna et al. observed an overlap between certain eating disorder traits (drive for thinness and interpersonal distrust) and orthorexia [22]. This finding was confirmed by Parra-Fernandez et al. [23] as they remarked that the highest negative correlation coefficient (−0.564, *p* < 0.01) was observed between the subscale “drive for thinness” and orthorexia. Moreover, Koven and Senbonmatsu noticed that two features of AN, body dissatisfaction and perfectionism symptoms increased as ORTO-15 score decreased [2].

### 3.3. Influence of Age/Gender

Considering the age parameter, subjects aged between 13 and 16 years old presented the highest risk of orthorexia [24]. That was also the finding in the study of Bona et al. where orthorexia was more likely to be observed in younger and rather fit participants [22]. Moreover, the risk of displaying orthorexia tendency as well as ED symptoms was induced by the present and past use of a special diet [25,26]. Gender might also be considered as a factor which might influence the occurrence of orthorexia, but there are still no clear results from studies [27,28].

### 3.4. Prognosis

As far as prognosis and features of EDs is concerned (Table 1/lower panels), Segura-Garcia et al. [11] showed that orthorexia symptoms were observed with high incidence among patients with AN and BN and tended to increase after treatment. Barthels et al. indicated that individuals with AN and pronounced orthorexic eating behavior chose to eat more often foods which are labeled as healthy [29]. “Autonomy” and “competence” was lower in AN patients who reported low orthorexic eating behavior and higher in individuals with AN and pronounced orthorexic eating behavior compared to [29]. Interestingly, results demonstrated that adults who had “normal” eating behavior had no risk of ON, while adults who had psychological and affective traits of eating disorders had ON [30].

## 4. Discussion

The present systematic review highlighted a correlation between the presence of eating disorders and orthorexia [18,27]. Higher eating disorders were significantly associated with higher tendencies and orthorexic behaviors [33]. High scores on the EAT (higher orthorexia behaviors) were significantly associated with orthorectic tendencies [16,33].

Specifically in adolescents it was found that those suffering from eating disorders had an increased risk of orthorexia [3,17,35]. Anorectic individuals are commonly preoccupied with the purity of their diet and tend to avoid certain foods which they consider to be safe. Eliminating food groups results in weight loss and nutritional deficiencies [44]. The association between AN and EDs raises concerns about whether orthorexia and anorexia nervosa are two overlapping conditions [50]. An Italian study by Dell’Osso et al. identified similarities between the two conditions and proposed that ON could be considered as an AN phenotype [51]. Adolescent orthorectics, develop common symptoms of anorexia [2]. Orthorectic and anorexic individuals have difficulty setting themselves in set-shifting. Thus, for example, individuals with orthorexia usually develop specific rules for choosing food that cannot be with preservatives, rules for preparing food (such as eating only raw foods), and they have to eat alone or in specific conditions. These rules gradually increase in number and complexity, so the orthorectic person devotes more time and energy to follow these rules. This behavior and cognitive rigidity also occur in AN [52]. In addition, orthorexia was more strongly associated with the symptoms of AN and BN sharing common symptoms such as “goal-driven” direction, “ego-syntonic” coordination, or “positively reinforcing” character with dietary restrictions [42,44]. Both ON and AN/BN are distinguished by firm diets in relation to the nutritional properties of food and a strong dread of long-term effects of the food they fear [47]. On the contrary, ON seems to be a separate condition from Avoidant/Restrictive Food Intake Disorder (ARFID). Unlike ARFID, people with ON might prefer not to limit their intake linked to an interest in food, the sensory properties of what they eat, or because of a previous deterrent food experience, but because of a tenacity to be as healthy as possible. While these people with AN can suffer serious medical consequences due to their food choices, people with ON seem not to have any problems with perceiving their weight or body shape while their self-esteem is also not affected by weight or shape.

About potential causal associations, a lot of individuals presenting a risk of orthorexia and disordered eating habits, have stated that previous diet followed their disorder [44]. It appears that people who monitor their caloric intake and are afraid of gaining weight have higher rates of eating disorders, whereas those who care about proper nutrition and are concerned about weight gain had higher scores on orthorexia [17]. People who are trained in healthy eating major report that they are interested in patients and themselves, which can lead to food obsession [53]. Thinking about how food is being prepared or spending more time thinking about food and healthy eating can be risk factors that can lead to eating disorders [54].

Regarding associations with specific groups of youth, the study by Arslandas et al. found that nursing students were 84.5% more likely to develop an eating disorder and 45.3% of the same students were at risk of developing orthorexia [17]. Aksoydan and Camcı in their research highlighted the prevalence of orthorexia between opera singers and ballet dancers [55]. Of the total sample, 56.4% were identified at risk of orthorexia. Ergin [56] conducted a study on a group of healthcare staff and observed that 60.1% of them were at risk of ON. Nutrition students in a German study had higher scores on dietary restriction than students in other curricula. This restriction concerned the tendency for moderate food intake either for weight loss or for weight maintenance [57]. Nutrition students had higher rigid control and higher flexible control of eating behavior compared to the characteristic control group that may cause some concern.

It is ascertained that orthorexia seems to be different from AN and BN. In ON, dietary restrictions are not driven by excessive fear of weight gain, or by the excessive effect that shape and weight have on their self-assessment, nor by distorted body image [58]. Body image dissatisfaction is mostly observed in other eating disorders including binge eating [59]. While overeating and cleansing or inadequate calorie compensation contributes to symptoms of anorexia and orthorexia, they are not part of the recommended ON diagnostic picture [58]. These distinctions are important, as traditional therapeutic approaches and follow-up to eating disorders such as anorexia may not be appropriate for people with ON [58]. Orthorexia and psychogenic anorexia and bulimia are associated with intense obsessions and compulsive behaviors, which link these conditions to obsessive-compulsive disorder, such as repetitive, intrusive thoughts about food and health at inappropriate and inappropriate times and a strong need to organize food in a ritual way [60]. Similar to OCD people, orthorectic people have limited time for other activities, as adhering to a strict diet affects their normal routines [13].

According to the available literature, most of the young adults who have been diagnosed with any kind of eating disorders and have received treatment in specialist eating disorder services present great results at long-term follow-up; however, even after many years, a significant number still suffer from other mental health problems, requiring therapeutic approach [61]. Nevertheless, the frequency of follow-up does not seem to reduce nor the risk of nutritional deficiencies or the use of dietary supplement [62].

The results of the present systematic review should be interpreted with some concern due to the limitations of the eligible studies. One of those limitation is the cross-sectional design, which does not permit establishment of a causal relationship between disorder eating and ON, as well as the fact that a variety of questionnaires was used. Moreover, another limitation is the low quality ratings [2,3,11,16,17,18,19,20,21,22,23,24,25,26,27,28,29,30,31,32,33,34,35,36,37,38,39,40,41,42,43,44,45,46,47,48,49]. Furthermore, factors such as the small sample in some studies, the lack of subgroup data about bulimia, and the lack of a universally accepted instrument to assess orthorexia should be taken into consideration. The fact that only studies in English were included may have limited generalizability of findings to countries with a western lifestyle.

## 5. Conclusions

In conclusion, this systematic review indicated a potential association between orthorexia and eating disorders. Further research is needed to evaluate notions of causality, as well as to document factors signaling similarities and discrepancies between these conditions.

## Figures and Tables

**Table 1:** Demographic characteristics of eligible studies. Studies performed exclusively on adolescents and young adults are presented in the upper panels, while studies with admixture with older individuals are presented in the middle panels. Studies concerning prognosis and features of eating disorders are presented in the lower panels.

Author (Year)	Region, Country	Study Period	Study Design	Sample Size	Percentage of Males	Mean Age (SD)	Age Range	Study Population	Associations Studied in Relation to Orthorexia	Definition of Orthorexia	Definition of Eating Disorders	Main Findings of the Study	Potential Cofounding Factors Assessed	Nos Quality Rating
Studies exclusively on adolescents and young adults														
Agopyan 2018 [16]	Turkey (Istanbul)	March to May 2017	Cross-sectional	136	0	20.9 ± 2.0 years	N/R	College students	Eating disorder	ORTHO-11 > 27	EAT-40 > 30	A significant negative correlation (*p* < 0.05) was observed between the EAT-40 and ORTO-11 scores.	None	5/10
Arslantas 2017 [17]	Turkey (Izmir)	May 2018	Cross-sectional	181	22	N/R	N/R	College students	Eating disorder	ORTHO-11 *	EAT-40 > 30	A low negative correlation was identified between EAT-40 and ORTHO-11 (r = −0.162; *p* = 0.027). 84.5% were at risk according to EAT40, and 45.3% were at risk of ON	None	5/10
Brytek-Matera 2017 [27]	Italy	N/R	Cross-sectional	120	30.8%	22.74 ± 7.31 years	19 to 30	College students	Eating disorders	ORTHO-15 < 35	The Eating Attitudes Test-26 (EAT-26) *	In female students a statistically significant positive, but weak, correlation was found between ORTO-15 and body image discomfort (r = 0.39 and r = 0.32; *p* = 0.001 and *p* = 0.003) Concerning, male students as the scores decreased on the ORTO-15 (more pathologica), the EAT-26 scores were linked to healthier eating attitudes. No significant differences were observed between females and males	Νone	5/10
Bundros 2016 [31]	USA	Academic year of 2014 to 2015	Cross-sectional	448	27	22.17 ± 4.83	N/R	College students	Eating disorders	Bratman Orthorexia Test (BOT) *	Eating Attitude Test-26 (EAT-26) *	Significant positive correlations were discovered between total BOT and EAT-26 scores (r = 0.47, *p* < 0.01). Higher orthorexic tendencies were associated with higher eating disorder risk	None	5/10
Dunn 2019 [32]	USA	Ν/R	Cross-sectional	354	10	21.6 years (SD 12.9)	N/R	College students and clinical cases	Eating disorder	ORTHO-15 *	Eating Attitude Test (EAT-26) *	The mean EAT-26 score for those indicated to have ON was in the range for having possibly an eating disorder.	None	6/10
Farchakh 2019 [33]	Lebanon	Between May 2018 and January 2019	Cross-sectional	627	50	21.81 ± 2.00	N/R	Medical students	Eating disorder	ORTHO-15 *	The Eating Attitude (Test-26) *	Higher EAT score (β = −0.094) was significantly associated with lower ORTO-15 scores	BMI, age, gender	7/10
Fidan 2010 [28]	Turkey	January 2008	Cross-sectional	878	53	21.3 ± 2.1 years	16 to 29	Medical students	Eating disorders	ORTHO-11 *	Eating Attitude Test-40 > 30	Male students presented a statistically significantly higher tendency for orthorexia (*p* = 0.001). A statistically significant difference for tendency for orthorexia was also observed between the different age groups (*p* = 0.025). In logistic regression analysis, age, sex, Eating Attitude Test-40 (EAT-40), and height affected the ORTO-11 scores. The difference between mean ORTO-11 scores of the groups with EAT-40 scores of 30 or lower was statistically significant (*p* = 0.028)	None	5/10
Hayles 2017 [34]	USA	Ν/R	Cross-sectional	404	17	20.71 (SD 5 4.36)	N/R	Undergraduate students	disordered eating	Bratman Orthorexia Self-Test *, ORTHO-15 *	Eating Disorder Examination Questionnaire *	Orthorexia symptoms presented small to medium correlations with disordered eating symptoms. Orthorexia nervosa symptoms demonstrated an inverse and statistically significant relationship with the total EDEQ score.	None	6/10
Koven 2013 [2]	USA	N/R	Cross-sectional	100	21	19.3 (1.2)	18 to 22	College students	Neurophysiological functioning	ORTHO-15 *	Eating Disorder Inventory (EDI-2) *	Body Dissatisfaction and perfectionism symptoms, as features of AN augmented as ORTO-15 score was reduced, meaning that orthorexia severity was higher	Age, education, and estimated intellectual functioning	7/10
Lucka 2019 [24]	Poland	N/R	Cross-sectional	864	30	13–30 (males) 18–29 (females)	13–29 (women) 13–30 (men)	Secondary and tertiary students	Eating disorders	ORTO-15 < 40	EAT-26 *	The highest risk of orthorexia was found in junior secondary school students, aged between 13 and 16 years old The lowest risk was observed in senior secondary school (16 to 19-years old) Individuals at risk of orthorexia scored higher in the EAT-26, (EAT-26 total score z = 9.90; *p* < 0.001) (EAT bulimia: r = 0.259 *p* < 0.001)	None	5/10
White 2020 [35]	United States	N/R	Cross-sectional	103	100%	19.84, SD = 1.71	18–25	Undergraduate students	Eating disorder pathology	ORTO-7 *	The Eating Disorder Diagnostic Scale (EDDS) *	Orthorexia symptoms presented a positive association with other dimensions of eating pathology (r = 0.535, *p* < 0.001)	None	6/10
Studies on adolescents and young adults, with admixture with older individuals														
Asil 2015 [36]	Turkey (Ankara)	December 2012– March 2013	Cross-sectional	117	14	34 ± 11.2 years	N/R	Dieticians	Eating disorder	ORTHO-15 *	EAT-40 > 30	The participants who had ORTO-15 scores less than 40 points presented significantly higher scores for EAT-40 (*p* < 0.05). A significant negative correlation was observed between the ORTO-15 and EAT-40 score	None	5/10
Barnes 2016 [37]	Australia	2017	Cross-sectional	220	21	23.81 (8.40)	18 to 62	college students	History of eating disorder and personality traits	ORTHO-15 < 36	Eating disorder (anorexia of bulimia neurosa) was self-reported	The most significant predictor of orthorexia nervosa was the presence of an eating disorder history. A significant difference was found on the ORTO-15 score between those having a positive history of eating disorder (M = 17.94, SD = 1.01) and those who did not (M = 23.37, SD = 4.20), t (159) = −5.15, *p*0.001.	None	5/10
Bona 2019 [22]	Hungary	March 2017–October 2017	Cross-sectional	207	32	31.9 (8.7)	18 to 59	Gym attendees	Eating disorder	Orto-11-Hu *	Eating Disorder Test (EAT-40) *	(Drive for thinness and interpersonal distrust was the two characteristics that presented significant associations with higher orthorexic tendencies. A relation to age was indicated: younger participants were more likely to have orthorexic habits.	Eating Disorder Inventory, health and exercise habits, and demographics	8/10
Brytek-Matera 2020 [38]	Spain and Poland.	N/R	Cross-sectional	860	34.9%	21.17 ± 3.38	18–35	University students	ED symptoms	Düsseldorf Orthorexia Scale (DOS) *	Eating Disorder Inventory (EDI) *	DOS scores and bulimia were significantly correlated (r = 0.170; *p* < 0.01)	None	5/10
Gramaglia 2016 [39]	Italy, Poland and Spain	1 January 2016 to 31 December 2017	Cross-sectional	664	28.00%	24.02 years	17–54	University students	Eating patterns	ORTO-15 < 35 was used for Italy and Spain, and the cutoff of 24 for Poland	Eating Attitudes Test-26 (EAT-26) *	More than a third of the sample presented ON, F	Gender	6/10
Haddad 2019 [18]	Lebanon	Between January and May 2018	Cross-sectional	811	34	27.59 ± 11.76	N/R	Lebanese governorates	Eating habits	ORTHO-15 < 40	Eating Disorder Inventory (EDI-2), Eating attitudes test (EAT-26) > 21	Increased EAT scores (Beta = −0.087) were related to lower ORTHO-15 scores, meaning higher level of orthorexia tendencies and behaviors	Sociodemographic Factors	8/10
Kyle 2018 [40]	USA	N/R	Cross-sectional	124	12.10%	21.3 ± 3.1 w	18 to 34	College students and yoga attendants	Eating patterns	ORTO-15 < 40	Obsessive–Compulsive Inventory (OCI-R) > 21	No significant relationship was found between a history of an eating disorder and ON [X 2 (1, N = 124) = 0.481, *p* = 0.488)] or possible eating disorder [X 2 (1, N = 124) = 0.393, *p* = 0.531	Gender, age	7/10
Łucka [3]	Gdansk and Gdynia	N/A	Cross-sectional	864	N/A	20.21 +/− 3.27 females, 18.93 +/− 3.67 males	13–30	Students	Eating attitude	ORTO-15 < 35	EAT-26 > 20	27% of subjects were reported to have a great risk of ON. This risk was significantly higher in individuals with ED, both women (χ^2^ = 58.300; *p* < 0.001) and men (χ^2^ = 6.376; *p* = 0.012)	None	5/10
Mahesh Shah 2012 [19]	USA	Spring of 2010	Cross-sectional	172	41	19.83 (SD = 2.73 range: 18–39)	18 to 39	College students	Eating disorders	ORTHO-15 < 40	EAT-26 > 20	A significant negative correlation was identified between disordered eating behaviors and ON (r = −0.33, *p* < 0.01)	None	6/10
McInerney-Ernst 2011 [21]	USA	2010	Cross-sectional	163	42	(M = 19.94, SD = 3.01)	18 to 40	College students	Eating disorder	ORTHO-15 < 40	Eating Disorder Examination Questionnaire (EDE-Q) *	Self-reported disordered eating patterns significantly predicted ON symptoms. Fewer ON symptoms were related to higher level of disordered eating pattern. Regression analysis results were statistically significant, F (1, 159) = 11.32, *p* = 0.001. Disordered eating patterns (EDE-Q; β = −0.26) was the only significant predictor of the ORTO-15 component measuring eating concern and worry.	None	5/10
Obeid 2021 [41]	Lebanon	October 2020	Cross-sectional	787	22.30%	23.87 ± 8.75	>18	Community dwelling participants	Eating disorders	ORTO R *	Eating Attitude Test (EAT 26) > 20	More ON tendencies and behaviors were associated with, higher EAT scores (β = 0.15)	Difficulties in emotion regulation, alexithymia, (DERS score)	9/10
Okumuşoğlu 2017 [20]	Turkey	Ν/R	Cross-sectional	100	43	22.35, SD: 2.194	18 to 60	College students	Eating disorders	ORTHO-11 *	Eating attitude test-40 (YTT-40) > 30 and REZZY eating disorders scale (REZZY) > 2	ED tendencies which were measured by REZZY scores presented a negative correlation with ORTO-11 scale (r = −0.328, *p* = 0.01) but not with YTT-40 scores	None	5/10
Parra-Fernández 2018 [23]	Spain	N/R	Cross-sectional	454	35	21.74 ± 4.73 years (18–51)	18 to 41	College students	Eating disorders	ORTO-11-ES < 25	Eating Disorder Inventory-2 questionnaire (EDI-2) *	High values of the ED subscales corresponded to low values for the ON scores. The highest (negative) correlation coefficient (−0.564, *p* < 0.01) was observed between drive for thinness and the ON score. Significant were the scores on the EDI-2 for the group at risk of ON regarding their drive for thinness (17.1% vs. 2.1%), bulimia (2.6% vs. 0%) and body dissatisfaction (26.3% vs. 12.4%)	None	6/10
Parra-Fernández 2019 [42]	Spain	2017/2018	Cross-sectional	492	43.1%	19.97 years (SD = 3.03)	18 to 44	College students	Compare the prevalence of ON	Düsseldorfer Orthorexie Skala (DOS-ES) > 30 ORTO-11-ES < 25	The Eating Disorder Inventory-EDI-2-Spanish Version *	ORTHO and bulimia were significantly correlated (t (1, 489) = 4.19, *p* < 0.01).	None	6/10
Plichta 2017 [25]	Poland	2017	Cross-sectional	1120	29.6%	N/R	18–35	students	The dietary patterns (DPs) of people showing ON tendency, ED symptoms, and both ON tendency and ED symptoms	ORTO-15 < 35	Eating Disorder Screen for Primary Care (ESP) *	Both ON tendency and ED symptoms were reported in 13% of the sample. The use of a special diet in both past and present might lead to an increased risk of ON tendency and ED symptoms.	None	5/10
Segura-García 2012 [26]	Italy	From May 2009 to April 2010	Cross-sectional	577 athletes and 250 controls	67	23.2 ± 5.5 (males), 21.3 ± 7.0 (females)	16 to 45	Athletes of judged sports, team sports and fitness activities and controls from college students	Eating disorder	ORTHO-15 < 35	Eating Attitude Test 26 (EAT-26) > 20, and Yale-Brown-Corner Eating Disorder Scale (YBC-EDS) > 8.	Age, diet in the past positivity to YBC-EDS or to EAT-26, competition level, and number of YBC-EDS preoccupations/ rituals were considered to be independent predictors of ON	Age, gender, BMI, activity level, professional competitive level, previous dieting, actual dieting, EAT-26 positivity, YBC-EDS positivity, BUT positivity, YBC-EDS symptoms	7/10
Strahler 2018 [43]	Germany	February to April 2017	Cross-sectional	713	22	28.9 ± 10.6, 29.4 ± 11.2 years (range: 18–75 years, median: 25 years) = women	Women, 18–75	Population survey	eating disorder	Duesseldorf Orthorexia Scale (DOS) > 30	Eating Disorder Examination—Questionnaire (EDE-Q8) > 2.5	The pathological eating explained the highest percentage of variation in ON (R^2^ = 0.380). In order to discriminate ON from other mental health symptoms, a substantial co-occurrence with pathological eating with about 78% of ON subjects was suggested showing above-threshold symptoms of an eating disorder. Addictive behaviors were not related to ON.	None	5/10
Zickgraf 2019 [44]	USA	N/R	Cross-sectional	449	51	33.6 (9.5)	20 to 69	Internet population	Eating disorders	Eating habits questionnaire (EHQ) *	Clinical impairment assessment—Eating only (CIA-E) *, Eating attitudes test-Severe restricting for thinness/bingeing and purging (EAT-26-SRT/BP) *	ON symptoms were related more to AN/BN than to ARFID. Clinical impairment from eating was not related to overall ON symptomatology	Gender, BMI and weight	7/10
Studies on Prognosis/features of Eating Disorders														
Barthels 2016 [29]	N/R	N/R	Cross-sectional	72	0	M = 21.17, SD = 6.88 years (anorexia patients)	N/R	42 female patients diagnosed with anorexia nervosa and 30 female participants for control group	Analyze orthorexic eating behavior in anorexic individuals	Düsseldorf Orthorexie Skala (DOS) > 30	Female patients diagnosed with anorexia nervosa	No significant differences were observed between the AN group, the ANO and the control group. Patients with pronounced orthorexic eating behavior tend to eat more often healthy food, regardless of calorie content	None	5/10
Brytek-Matera 2015 [45]	Poland	From May 2014 to November 2014	Cross-sectional	52 female patients	0	22.81 years (SD = 3.80)	N/R	Outpatients at the Polish National Center for Eating Disorders.	Eating disorders	ORTHO-15 < 24	The Eating Attitudes Test-26 (EAT-26) > 20	ON was negatively predicted by eating pathology, weight concern, health orientation, and appearance orientation. Orthorexic behaviors were not found to be significant with pathways between other variables. Orthorexic behaviors were more frequent in the group with lower level of eating pathology and less common in the group reporting higher levels of eating pathology.	None	5/10
Brytek-Matera 2020 [30]	Poland	N/R	Cross-sectional	230	23.9%	26.52 ± 7.65	18–60	Participants from universities, companies and health centers	Pathological eating behaviors	The Düsseldorf Orthorexia Scale (DOS) > 30	The Three-Factor Eating Questionnaire (TFEQ-R18) *, The Eating Disorder Inventory (EDI) *	In the whole sample of adults, 3.0% presented traits of ON, 5.7% were at risk of ON, and 91.3% presented no risk of developing ON. A higher percentage of ON was observed in patients with high inappropriate eating behaviors, high psychological, and affective ED traits and moderate OCD features. Higher DOS scores presented significant associations with higher cognitive restraint, uncontrolled and emotional eating, drive for thinness and bulimia.	None	6/10
Gramaglia 2017 [46]	N/R	N/R	Cross-sectional	97	N/R	N/R	> 18 years	Patients with a diagnosis of AN	Orthorexic behaviors between clinical and non-clinical groups	ORTO-15 < 40	AN diagnosis	No difference was recorded (Chi-square test) in the percentage of subjects scoring under the ORTO-15 cutoff between Italian AN and Italian HC (*p* = 0.263), and between Polish AN and Polish HC (*p* = 0.670).	None	5/10
Kiss Leizer 2018 [47]	N/R	N/R	Cross-sectional	739	N/R	M = 29.67 SD = 10.18	18 to 72	Social media respondents	Personality profile	Ortho-11 *	Temperament and Character Inventory-56 (TCI-56) *	There was a difference recorded between the ON groups in harm avoidance (F (2, 736) = 16.32, *p* < 0.001, η2 = 0.04). A significant difference was also reported between ON groups on self-directedness factor (F (2, 736) = 19.16, *p* < 0.001, η2 = 0.05). High harm avoidance and low self-directedness are relevant factors of AN and BN	None	5/10
Sanlier 2016 [48]	Turkey	April and May 2014	Cross-sectional	900	42	20.37 ± 1.74	17–23	College students	Eating disorder	ORTHO-15 *	EAT-40 *	EAT-40 and ORTO-15 scores were significantly negatively correlated. Orthorectic participants among women were more than men (*p* < 0.001)	Gender	6/10
Segura-Garcia 2015 [11]	Italy	Ν/R	cross-sectional	32 patients with eating disorders and matched controls	0	22.2 ± 3.4 at the follow up	N/R	Eating disorders patients	eating disorders and OCD	ORTHO-15 < 35	Yale-Brown-Cornell Eating Disorder Scale (YBC-EDS) > 30 and EAT-26 > 20	ON highly prevalent symptoms among patients with AN and BN which tend to increase after treatment.	Age, gender, BMI	7/10
Yakın 2020 [49]	France	N/R	Cross-sectional	921	15.3%	20.72 (SD = 2.63)	18 to 30	Students	ED behaviors	Eating Habits Questionnaire (EHQ) *	Eating Disorders Inventory-3rd Edition (EDI-3) *	“Orthorexic behavior” and “Eating disordered behavior” clusters displayed greater appearance orientation and overweight preoccupation compared to the “Low” cluster, could be interpreted as an important resemblance between ON and ED.	None	5/10

* AN: anorexia nervosa; CI: confidence interval; EAT: Eating Attitudes Test; EDI: Eating Disorder Inventory; EDI-DT: Eating Disorder Inventory “Drive for Thinness” subscale; EDE-Q: Eating Disorder Examination-Questionnaire; ESP: Eating Disorder Screen for Primary Care; NOS: Newcastle-Ottawa Scale; NR: not reported; OCI-R: Obsessive–Compulsive Inventory-Revised; ON: Orthorexia Nervosa; TCI-56: Temperament and Character Inventory-56; TFEQ-R18:TheThree-Factor Eating Questionnaire R-18; YBC-EDS: Yale-Brown-Cornell Eating Disorder Scale; ANO: anorexia with pronounced orthorexic eating behavior.

## Data Availability

Data are included in the manuscript.
